# Molecular Epidemiology of Vancomycin-Resistant Enterococci Bloodstream Infections in Germany: A Population-Based Prospective Longitudinal Study

**DOI:** 10.3390/microorganisms10010130

**Published:** 2022-01-08

**Authors:** Carlos L. Correa-Martínez, Annette Jurke, Janne Schmitz, Frieder Schaumburg, Stefanie Kampmeier, Alexander Mellmann

**Affiliations:** 1 Institute of Hygiene, University Hospital Münster, 48149 Münster, Germany; Janne.Schmitz@ukmuenster.de (J.S.); Stefanie.Kampmeier@ukmuenster.de (S.K.); Alexander.Mellmann@ukmuenster.de (A.M.); 2 Section Infectious Disease Epidemiology, North Rhine-Westphalian Centre for Health, 44801 Bochum, Germany; Annette.Jurke@lzg.nrw.de; 3 Institute of Medical Microbiology, University Hospital Münster, 48149 Münster, Germany; Frieder.Schaumburg@ukmuenster.de

**Keywords:** vancomycin-resistant enterococci, VRE, bloodstream infections, molecular epidemiology, Germany, Europe

## Abstract

Vancomycin-resistant enterococci (VRE) pose a public health challenge worldwide. While VRE bloodstream infections (VREBI) increase in Germany and Europe, population-based molecular data are scarce. We aimed to analyze the molecular epidemiology, demographic aspects, and geographical distribution of VREBI in the German Federal State of North-Rhine–Westphalia (NRW), located in the German–Dutch–Belgian border area, representing over 20% of Germany’s population. VREBI isolates were collected from hospitals across NRW between 2016 and 2019. Demographic data were gathered and anonymized upon sample collection. Multilocus sequence typing (MLST) and identification of glycopeptide resistance were carried out. Epidemiological analysis and geographical mapping were performed. Single VREBI isolates from 755 patients were analyzed. In total, 38.9% were female, and 80.0% were aged ≥ 60 years. The VREBI incidence per 100,000 inhabitants nearly tripled, from 0.52 (2016) to 1.48 (2019), particularly in male patients aged ≥ 50 years. The proportion of *vanB* reached 83% (*n* = 202/243) in 2018, overtaking *vanA* as the predominant glycopeptide resistance determinant, detected in close relation with ST117 isolates. The proportion of MLST sequence type (ST) 117 peaked in 2018, at 78.2% (*n* = 190/243). The major role of these emerging strains in invasive infections in central Europe requires novel strategies for their diagnosis, treatment, and prevention.

## 1. Introduction

Since their first description three decades ago [1,2], vancomycin-resistant enterococci (VRE) have become widespread, top-priority, multidrug-resistant organisms (MDRO) [3]. Though commonly found as intestinal commensals, enterococci are capable of causing a wide range of infections, including abdominal, surgical site, and foreign body-associated infections, as well as endocarditis [4]. VRE bloodstream infections (VREBIs) are associated with mortality twice as high as that of vancomycin-sensitive enterococci bloodstream infections (VSEBIs) [5]. This translates into increased hospital costs, excess mortality, and prolonged hospital stays [6,7]. In Europe, the proportion of VRE among bloodstream infections caused by *Enterococcus faecium* has substantially increased in recent years (2015, 10.5% vs. 2019, 18.3%) [8]. Within this period, VREBIs have increased even more markedly in Germany, from 10.5% to 26.3% [8].

Phenotypic resistance against glycopeptides (e.g., vancomycin, teicoplanin), is genetically encoded by resistance determinants designated as *van*-operons. These comprise several variants and may be located either in chromosomal regions or in mobile genetic elements [9], rendering them transmissible among enterococci but also among other microorganisms (e.g., staphylococci) [10]. In the context of human infection, *vanA* and *vanB* are the most relevant resistance determinants, encoding a combined resistance against vancomycin and teicoplanin or solely against vancomycin, respectively [9]. In the past few decades, *vanA* has predominated over *vanB* in Europe, Asia, and the Americas, with *vanA*-positive strains accounting for over 89% of all VRE isolates [11,12,13,14,15]. An increase in the incidence of *vanB*-positive VRE isolates has been described recently in studies carried out in Europe [16,17]. In Germany, where *vanA*-positive strains have been historically most prevalent, the German National Reference Centre (NRC) for Staphylococci and Enterococci reported a shift in the *vanA*/*vanB* ratio for the first time in 2016 [18] and confirmed an increasing trend of *vanB* in the following years [19]. Studies carried out in different German hospitals within the past two years indicate a predominance of *vanB*-positive VRE strains, mainly collected from rectal swabs upon admission [20,21] or in hospital outbreaks [22]. However, detailed population-based data on VREBIs are scarce due to the lack of mandatory notification in Germany. Official statistics on the proportion of VRE among enterococci in Europe are published yearly by the European Centre for Disease Control and Prevention [8]. These data are based on invasive infections (blood and cerebrospinal fluid) as benchmarks that facilitate comparison between countries. Prospectively collected information on the molecular epidemiology of such invasive VRE infections at a population level is currently not available. We therefore performed a comprehensive population-based survey on the molecular epidemiology of VREBIs in Germany.

## 2. Materials and Methods

In cooperation with local public health offices, a state-wide network of 31 microbiology laboratories (Table S1) in charge of processing blood culture samples from hospitals across North-Rhine–Westphalia (NRW) was established. NRW, located in the German–Dutch–Belgian border region, constitutes the most populous German Federal State, with over 20% of the total German population (Figure S1).

All patients diagnosed with VREBIs (nosocomial and community acquired) within the study area were included. Isolates were collected between 1 January 2016 and 31 December 2019. Only the first received isolate from each patient was analyzed. The study area comprised healthcare facilities in any of the 53 administrative districts (Figure S1) of NRW.

### 2.1. Demographic and Geographic Analyses

Information on the sex and age of patients was collected directly at the treating hospital and anonymized before sample submission for molecular analysis. Regional demographic data were obtained from databases made available by NRW’s statistical office (https://www.landesdatenbank.nrw.de/ldbnrw/online/data?operation=table&code=12411-01i&levelindex=0&levelid=1597225839095#astructure, accessed on 17 January 2021). Incidence was calculated for each year of the study period employing the mid-year population of NRW and each of the districts from which VRE isolates were received. To visualize the geographical distribution of the VREBI cases, results were mapped according to the location of the treating hospitals in the different administrative districts.

### 2.2. Molecular Analysis

Sequence types (STs) of VRE strains were determined employing a multilocus sequence typing (MLST) scheme, performing amplification of housekeeping genes by PCR and subsequent sanger sequencing of the PCR products, as previously described [23]. Obtained sequences were analyzed with SeqSphere^+^ software version 7.0.1 (Ridom GmbH, Münster, Germany). Glycopeptide resistance determinants were identified using a qPCR assay, the GenoType Enterococcus^®^ line probe, according to the manufacturer’s instructions (Hain Lifescience, Nehren, Germany).

### 2.3. Data Processing

Data were processed using the Stata statistical software version 16 (StataCorp, College Station, TX, USA). Categorical data (e.g., sex) were compared using the chi-squared test.

## 3. Results

In total, 755 isolates were included in the study (Table S2). The number of samples increased continuously from 93 (2016) to 154 (2017), 243 (2018), and 265 (2019).

### 3.1. Demographic Characteristics

**Figure 1:**
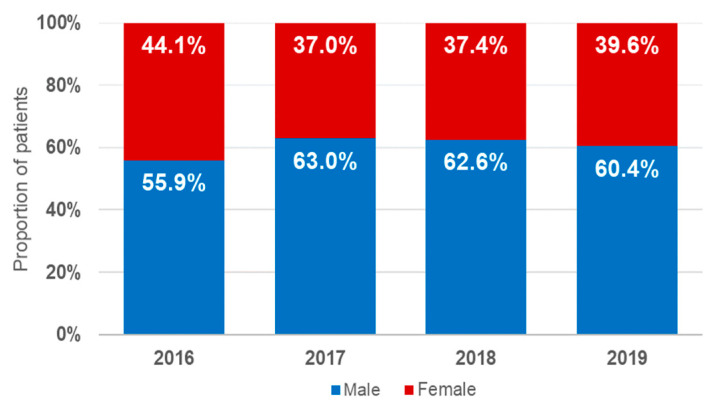
Sex distribution of the analyzed VREBI samples.

Male patients were significantly more affected by VREBIs (*p* < 0.001), accounting for 61.1% (95%CI: 59.3–62.9%) of the subjects included, while females represented 38.9% (95%CI: 37.1–40.7%) of the group. Sex distribution did not vary significantly over the course of the four-year study period (Figure 1).

**Figure 2:**
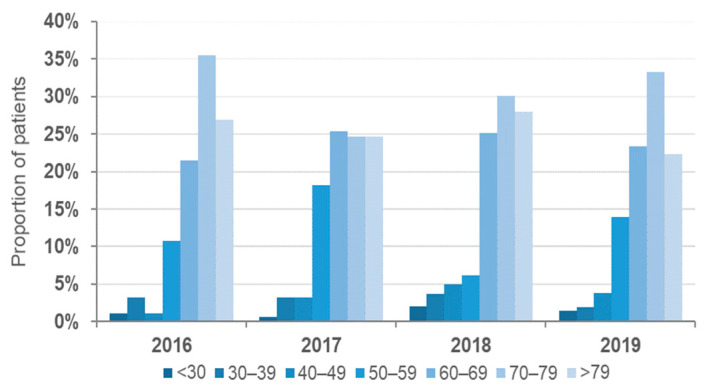
Age distribution of the analyzed VREBI samples.

Age groups most affected by VREBIs were 70–79 years (30.7%, *n* = 232/755); >79 years (25.2%, *n* = 190/755); 60–69 years (24.1%, *n* = 182/755). Up to 80% of all analyzed VRE strains were isolated from patients aged ≥ 60 years. No significant variation was observed in the age distribution throughout the study period (Figure 2). NRW’s 10 most populated cities (Figure S1b) concentrated 40.5% of all analyzed cases.

### 3.2. Molecular and Epidemiological Characteristics

All VRE isolates were identified as *E. faecium,* and no *E. faecalis* strains were detected. MLST analysis revealed 21 different STs. The five most common were ST117 (69.4%, *n* = 524/755), ST80 (15.2%, *n* = 118/755), ST203 (5.3%, *n* = 40/755), ST192 (2.8%, *n* = 21/755), and ST17 (1.3%, *n* = 10/755). *vanB* was identified in 71.4% (*n* = 539/755) of the samples, while *vanA* was detected in the remaining 28.6% (*n* = 216/755). No coexistence of both resistance determinants was found.

**Figure 3:**
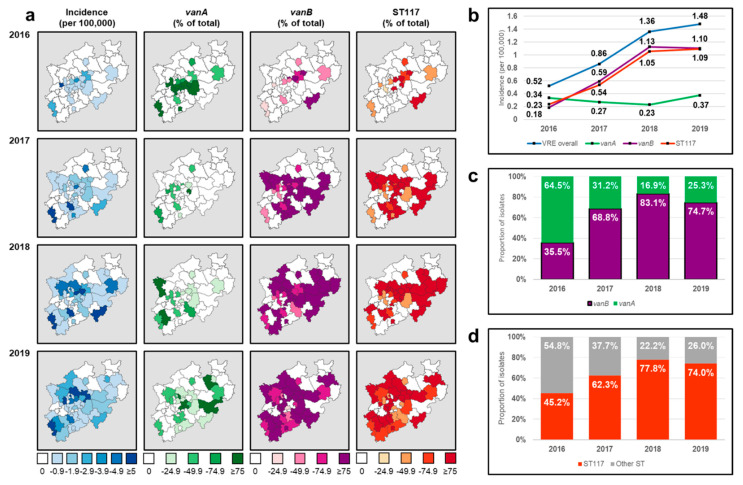
Temporal and spatial spread of VREBIs in NRW between 2016 and 2019: (**a**) geographic spread of VREBIs across administrative districts; (**b**) evolution of VREBI incidence; (**c**) distribution of resistance determinants; (**d**) proportion of ST117.

Raw incidence of VREBIs in NRW (per 100,000 inhabitants) rose continuously, from 0.52 in 2016 to 1.48 in 2019, showing a biphasic pattern: It increased by 162% between 2016 and 2018 and by 54% between 2018 and 2019 (Figure 3a,b). The number of districts submitting VREBI samples increased over the study period from 20 (2016) to 31 (2017), 40 (2018), and 44 (2019). The geographical distribution of the observed VREBI increase (Figure 3a) followed an east–west pattern across NRW.

The distribution of glycopeptide resistance determinants significantly varied over time. In 2016, *vanA* (64.5%, *n* = 60/93) predominated over *vanB* (35.5%, *n* = 33/90). In 2017, a clear inversion of these proportions was observed (*vanA*: 31.2%, *n* = 48/154; *vanB*: 68.8%, *n* = 106/154. This *vanB* predominance continued over the following months, peaking in 2018 at 83.1% (*n* = 202/243, Figure 3a,c). The incidence of *vanA* did not decrease in this period (Figure 3b).

The most commonly detected ST was ST117, with an increasing incidence throughout the study period (Figure 3a,b,d). In 2016, 45.2% (95%CI: 34.9–55.8, *n* = 42/93) of all isolates were identified as ST117, subsequently increasing until peaking at 78.2% (95%CI: 72.5–83.2, *n* = 190/243) in 2018. By 2019, this proportion represented almost three-quarters of the isolates collected that year (74%, 95%CI: 68.2–79.1, *n* = 196/265). The incidence of ST117 showed a development nearly identical to that of *vanB* (Figure 3b). The proportion of ST117 among *vanB*-positive strains increased continuously, from 75.8% (95%CI: 57.7–88.9%, *n* = 25/33) in 2016 to 94.4% (95%CI: 90.3–97.2%, *n* = 187/198) in 2019 (Figure 3d). Similarly, while 59.5% (95%CI: 43.3–74.4%, *n* = 25/42) of all ST117 strains carried the *vanB* operon in 2016, this proportion had reached 95.4% (95%CI: 91.5–97.9, *n* = 187/196) in 2019.

After ST117, the second most common ST was ST80, accounting initially for 19.4% (*n* = 18/93) of all isolates in 2016. This proportion decreased to 11.5% (*n* = 28/243) in 2018, finally reaching 18.9% (*n* = 50/265) in 2019. This followed the trend observed for the incidence of *vanA* isolates (Figure 3b), as 76.3% (*n* = 90/118) of all ST80 isolates carried the *vanA* resistance determinant.

### 3.3. Demographic Correlation of the vanB/ST117 Increase

**Figure 4:**
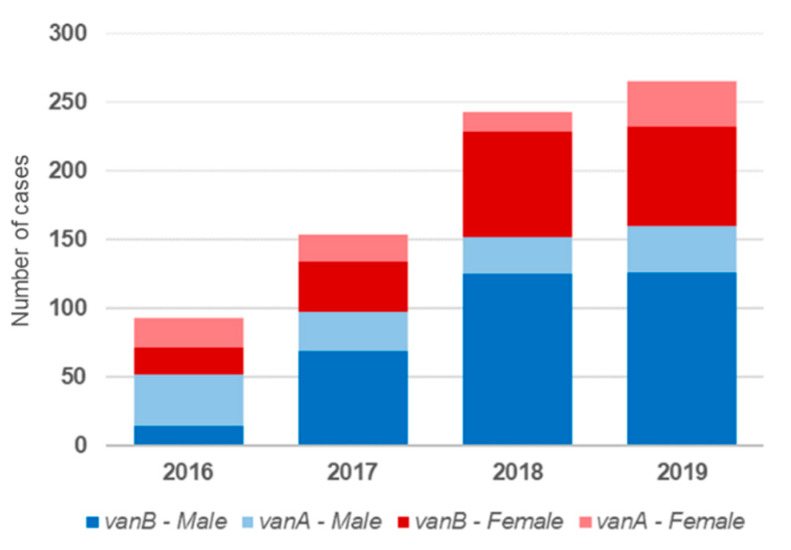
Sex distribution of the analyzed VREBI samples, classified by resistance determinants.

**Figure 5:**
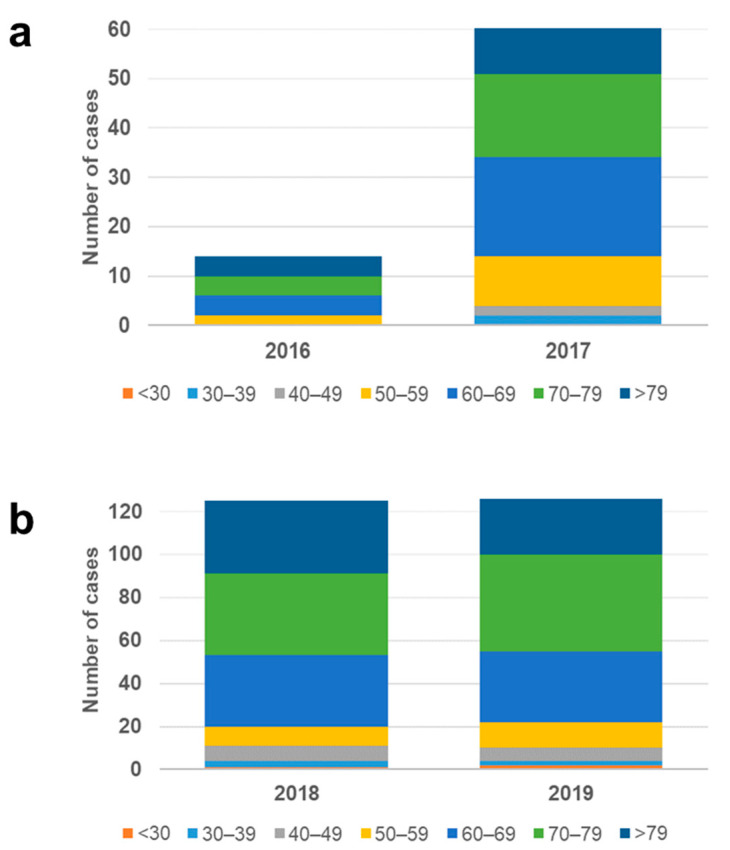
Age distribution of analyzed strains belonging to ST117, carrying *vanB* collected in (**a**) 2016–2017; (**b**) 2018–2019.

The sharp increase in the incidence of VREBI initially observed between 2016 and 2017 was determined by the occurrence of more bloodstream infections in male patients with *vanB*-positive strains (Figure 4), rising from 10.8% to 38.31% in this period. Within this subgroup, the proportion of VRE strains of ST117 rose from 71.4% to 85.5%, continuing to increase to 90% and 94% in 2018 and 2019, respectively. The majority of these patients belonged to the age groups ≥50 (Figure 5). The proportion of male patients aged ≥ 50 years, diagnosed with VREBIs caused by *van*B-positive ST117 strains, increased from 8.6% in 2016 to 35.7% of all analyzed isolates in 2017. A further increase was observed in 2018, reaching 42.4% and slightly decreasing to 41.1% in 2019.

## 4. Discussion

VRE poses an increasingly relevant challenge worldwide, given their ability to thrive in health care settings and cause life-threatening invasive infections. With a study population accounting for over 20% of Germany’s total inhabitants, this study constitutes the first population-based survey on the molecular epidemiology of VRE in central Europe. Here, we identified a significant increase in VREBI incidence over the study period and detected the time point at which the shift of the *vanA/vanB* ratio is believed to have occurred in Germany [18], and analyzed the further progression of this trend.

The overall VREBI incidence increased dramatically in NRW, from 0.48 to 1.48 per 100,000 inhabitants, between 2016 and 2019. This trend is in line with European data on VRE invasive infections [8,24]. The increase occurred following a biphasic pattern, rising sharply between 2016 and 2018, and more moderately between 2018 and 2019. Data from the German Antimicrobial Resistance Surveillance describe a higher VRE incidence in densely populated federal states, including NRW, suggesting a predominant occurrence in urban areas [25]. However, our analysis of the geographical distribution at the administrative department level in NRW revealed that the VREBI increase followed an east–west pattern over the study period. This does not correlate with the location of the densely populated Ruhr–Rhein metropolitan area and other densely populated urban centers in the state, distributed roughly around a northeast–southwest axis (Figure S1b). This indicates that VRE increasingly poses a challenge for health care institutions, in both urban and non-urban areas.

A remarkable change in the epidemiological panorama of VREBIs was observed in NRW in 2017, marked by an inversion of the *vanA* and *vanB* proportions among the analyzed isolates. For the first time, *vanB*-positive strains predominated in a region previously characterized by the high prevalence of VRE strains carrying the *vanA* operon. This coincides with reports of the German NRC describing a similar shift in the year 2016 [18]. This one-year delay in the occurrence of the *vanA/vanB* shift in VREBI isolates could be explained by the different sources of the strains analyzed. While all 193 isolates included in our study in 2017 originated from bloodstream infections, blood culture isolates represented only 5.1% (*n* = 95) of the samples analyzed by the NRC in 2016, with stool and rectal swabs, most likely from patients colonized but not infected, accounting for 43.5% (*n* = 808) of the samples [18]. Considering the natural progression of VRE colonization to infection, it is plausible that major changes in the molecular epidemiology of VRE are observed initially in colonized patients before becoming evident among patients with bloodstream infections. In 2018, the proportion of *vanB* strains was larger in NRW (81.1%), as compared with the NRC reports (68%) [26]. This discrepancy could indicate that VRE strains carrying *vanB* display an enhanced ability to cause bloodstream infections, as only 10.6% of all strains analyzed by the NRC were isolated from blood cultures [26].

In spite of the increasingly larger proportion of *vanB*-positive strains, the incidence of *vanA* isolates remained stable during the study period (Figure 3b). Therefore, the observed shift of the *vanA*/*vanB* ratio resulted solely from an increasing influx of *vanB* isolates, leading to speculation that these strains might have an enhanced capability to spread among susceptible populations, having achieved an overall expansion of the epidemiological niche of VRE in the region.

The incidence of ST117 increased sharply over the study period. While in 2016, this ST accounted for >50% of all analyzed isolates, the proportion increased continuously, until reaching approximately 75% in 2019. The overlapping trends in the incidence of *vanB*-positive strains and ST117 indicate the combined presence of these molecular characteristics in the strains accounting for the overall increase in VREBI cases. The proportion of ST117 isolates carrying the *vanB* operon increased from roughly 27% to 70% of all strains between 2016 and 2019. Thus, ST117 strains, currently the most commonly detected in Germany [26], seem to serve as vehicles for the *vanB* operon, facilitating its rapid spread.

Matched demographic and molecular data indicate that the drastic increase in VREBI cases observed within the study period was mainly determined by the occurrence of bloodstream infections among male patients aged ≥ 50 years, caused by *vanB*/ST117 strains. This is in contrast to German surveillance data (based mainly on VRE carriers), which show no sex predominance [25]. Assuming that these surveillance data are also representative of NRW, our data could indicate that colonization with *vanB*/ST117 VRE strains leads to VREBIs more commonly in male patients aged ≥ 50 years. This finding is relevant for risk profiling and improvement of empirical therapy of suspected enterococcal bloodstream infections in at-risk patients.

The main strengths of this work are the large study population, the regional relevance of the study area, as well as the inclusion of samples obtained exclusively from invasive infections. Due to its location, NRW constitutes a regional hub with dynamic border-crossing traffic in central Europe (Figure S1). Almost 15,000 people commute daily from The Netherlands and Belgium to NRW, and another 120,000 commute from the neighboring German federal states (https://www.it.nrw/70-jahre-amtliche-statistik-fuer-nordrhein-westfalen-jubilaeumsveroeffentlichung-mit-vielfaeltigen, accessed on 9 December 2021). Hence, the epidemiological relevance of NRW transcends its own borders and can thus be considered an indicator for Germany and neighboring countries. Given the controversy that continues to surround VRE and the need for special infection control measures to prevent their spread [27], data regarding their role in life-threatening infections are of major importance for the establishment of infection control policies in the context of an ever-rising incidence. Several studies have recently offered comprehensive insights into the molecular epidemiology of VRE in Germany, focusing, however, mainly on colonized patients [20,22,28]. Having exclusively analyzed isolates from bloodstream infections, our data illustrate the clinical relevance of VRE and the molecular characteristics of invasive strains, coinciding with reports of *vanB* increase with a predominance of ST117 in recent years.

The fact that sample submission by laboratories participating in the study was achieved on a voluntary basis and the lack of official statistics on the total incidence of VREBIs in Germany that allow the contextualization of our data represent the main limitations to this study. However, this represents a general problem related to the national notification strategies. A feasible strategy to tackle underreporting would be the mandatory notification of VREBI cases, such as in the case of invasive infections caused by methicillin-resistant *Staphylococcus aureus* [28].

In light of the observed emergence of *vanB* as a predominant resistance determinant, appropriate therapeutic options must be considered (e.g., teicoplanin) [9]. Aspects regarding diagnostics must also be considered. Data from Australia, where VRE epidemiology has been historically dominated by *vanB* strains, indicate that the lower degree of resistance of these strains against vancomycin can pose a challenge for the identification of VRE in clinical samples, potentially leading to the misclassification of resistance strains as sensitive [29].

## 5. Conclusions

The observed changes in VREBI resistance patterns and molecular distribution must be considered for the development of diagnostic, therapeutic, and prevention strategies in order to effectively tackle the increasing incidence of these invasive infections. The future development of trends in VREBI resistance patterns and molecular distribution warrant further prospective, continuous data collection, and interpretation.

## Data Availability

All data generated or analyzed during this study are included in this published article (and its Supplementary Information files).

## Supplementary Materials

The following are available online at https://www.mdpi.com/article/10.3390/microorganisms10010130/s1, Figure S1: (a) Location of NRW in Europe; (b) population density of NRW‘s administrative districts and location of the state‘s 10 most populated cities, Table S1: Laboratory network of the NRW vancomycin-resistant enterococci bloodstream infection molecular surveillance program, Table S2: Molecular and epidemiological data of VREBI isolates.
