# Supplementary material for: Molecular Epidemiology of Vancomycin-Resistant Enterococci Bloodstream Infections in Germany: A Population-Based Prospective Longitudinal Study
Source: Microorganisms. 2022 Jan 8;10(1):130. doi: 10.3390/microorganisms10010130 (PMC8777844; doi:10.3390/microorganisms10010130)
Supplement: Supplementary file 1 [file microorganisms-10-00130-s001.zip › Moelcular Epidemiology VRE_Supplementary Figure S1.pdf]

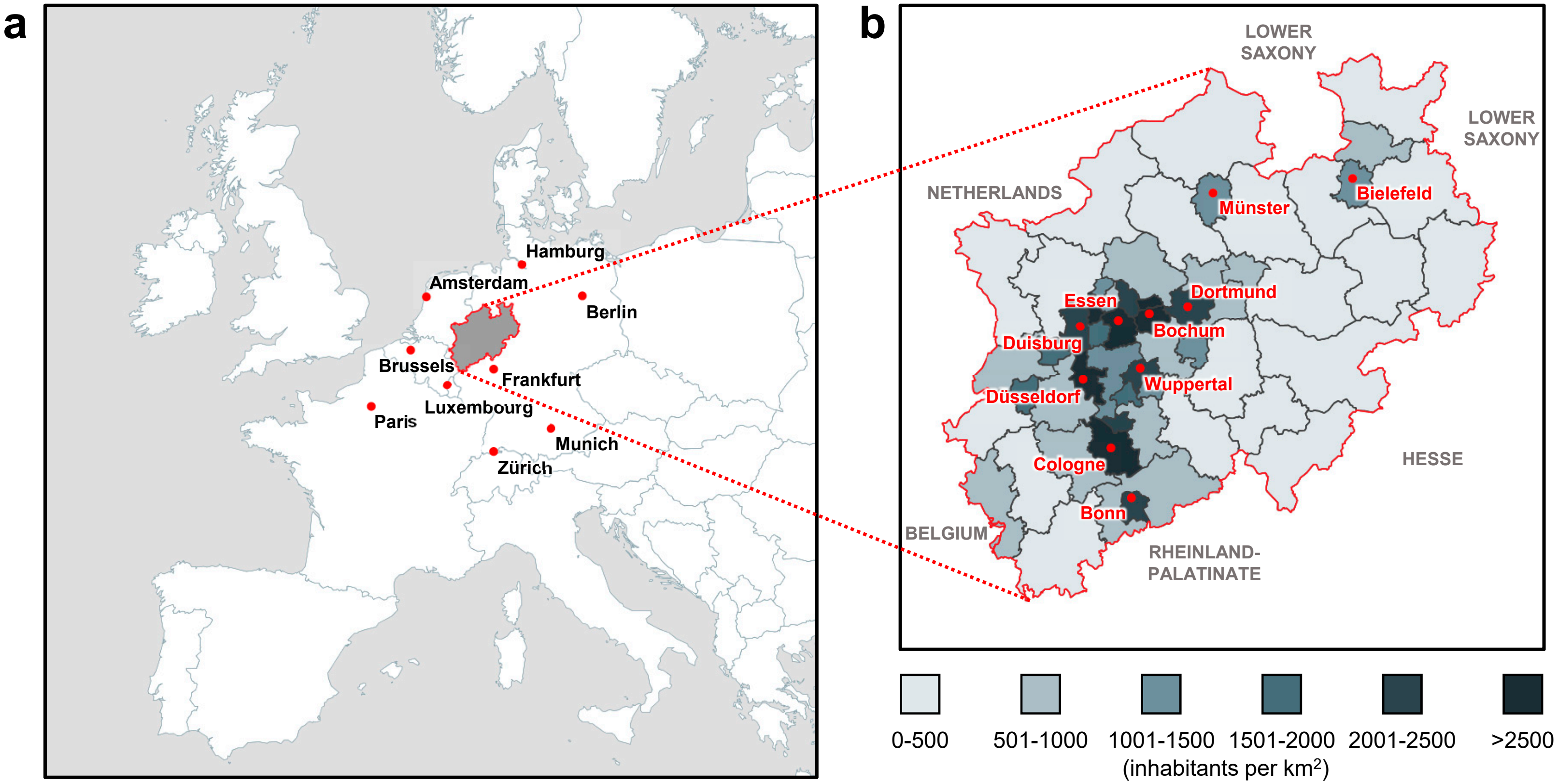

Figure S1. a) Location of NRW in Europe; b) Population density of NRW‘s administrative districts and location of the state‘s ten most populated cities.
